# PKG II Inhibits EGF/EGFR-Induced Migration of Gastric Cancer Cells

**DOI:** 10.1371/journal.pone.0061674

**Published:** 2013-04-16

**Authors:** Lu Jiang, Ting Lan, Yongchang Chen, Jianrong Sang, Yueying Li, Min Wu, Yan Tao, Ying Wang, Hai Qian, Luo Gu

**Affiliations:** 1 Department of Physiology, School of Medical Science and Laboratory Medicine, Jiangsu University, Zhenjiang, Jiangsu, China; 2 Department of Physiology, Nanjing Medical University, Nanjing, Jiangsu, China; University of Missouri-Columbia, United States of America

## Abstract

**Background:**

Our previous research results showed that Type II cGMP dependent protein kinase (PKG II) could block the activation of epidermal growth factor receptor (EGFR) and consequently inhibit the proliferation and the related MAPK/ERK-mediated signal transduction of gastric cancer cell line BGC-823, suggesting that PKG II might inhibit other EGFR-triggered signal transduction pathways and related biological activities of gastric cancer cells. This paper was designed to investigate the potential inhibition of PKG II on EGF/EGFR-induced migration activity and the related signal transduction pathways.

**Methodology/Principal Findings:**

In gastric cancer cell line AGS, expression and activity of PKG II were increased by infecting the cells with adenoviral construct encoding PKG II cDNA (Ad-PKG II) and treating the cells with cGMP analogue 8-pCPT-cGMP. Phosphorylation of proteins was detected by Western Blotting and active small G protein Ras and Rac1 was measured by “Pull-down” method. Cell migration activity was detected with trans-well equipment. Binding between PKG II and EGFR was detected with Co-IP. The results showed EGF stimulated migration of AGS cell and the effect was related to PLCγ1 and ERK-mediated signal transduction pathways. PKG II inhibited EGF-induced migration activity and blocked EGF-initiated signal transduction of PLCγ1 and MAPK/ERK-mediated pathways through preventing EGF-induced Tyr 992 and Tyr 1068 phosphorylation of EGFR. PKG II bound with EGFR and caused threonine phosphorylation of it.

**Conclusion/Significance:**

Our results systemically confirms the inhibition of PKG II on EGF-induced migration and related signal transduction of PLCγ1 and MAPK/ERK-mediated pathways, indicating that PKG II has a fargoing inhibition on EGF/EGFR related signal transduction and biological activities of gastric cancer cells through phosphorylating EGFR and blocking the activation of it.

## Introduction

Type II cGMP-dependent protein kinases (PKG II) is a serine/threonine kinase and accumulating research data indicated that this kinase had an important role in regulating cell biological activities such as proliferation and apoptosis, especially in tumor cells. In 2004, Cook *et al* found that PKG II could induce apoptosis of human cultured prostatic stromal cells [1]. In 2009, Swartling *et al* reported that PKG II inhibited proliferation of human neuroglioma cells and the inhibition was related to the decrease of the expression of transcription factor Sox9 and the phosphorylation of Akt [2]. In 2011, Fallahian *et al* found that cGMP could induce apoptosis of breast cancer cells and this effect was related to PKG II [3]. During our research, we found that the expression and the activity of PKG II in human gastric cancer cell lines were significantly lower than that of normal gastric mucosa cells [4]. Further study in our laboratory showed that PKG II could inhibit the proliferation of gastric cancer cell lines and block EGF induced signal transduction of MAPK/ERK-mediated pathway through preventing the activation of EGFR by EGF [5], [6]. Since the activation of EGFR can initiate several signal transduction pathways including MAPK/ERK, PI3K/Akt, JAK/STAT and PLCγ1-mediated pathways [7], [8], the blocking effect of PKG II on activation of EGFR suggests that this enzyme may have a wide-range inhibitory effect on signal transduction and the related biological activities of gastric cancer cells. This paper was designed to confirm this wide-range inhibitory effect of PKG II through investigating the inhibition of PKG II on EGF-induced migration activity and the related signal transduction in gastric cancer cells.

## Results

### PKG II Inhibits EGF-induced Cell Migration which is Associated with Signal Transduction of PLCγ1 and MAPK/ERK-mediated Pathways

**Figure 1:**
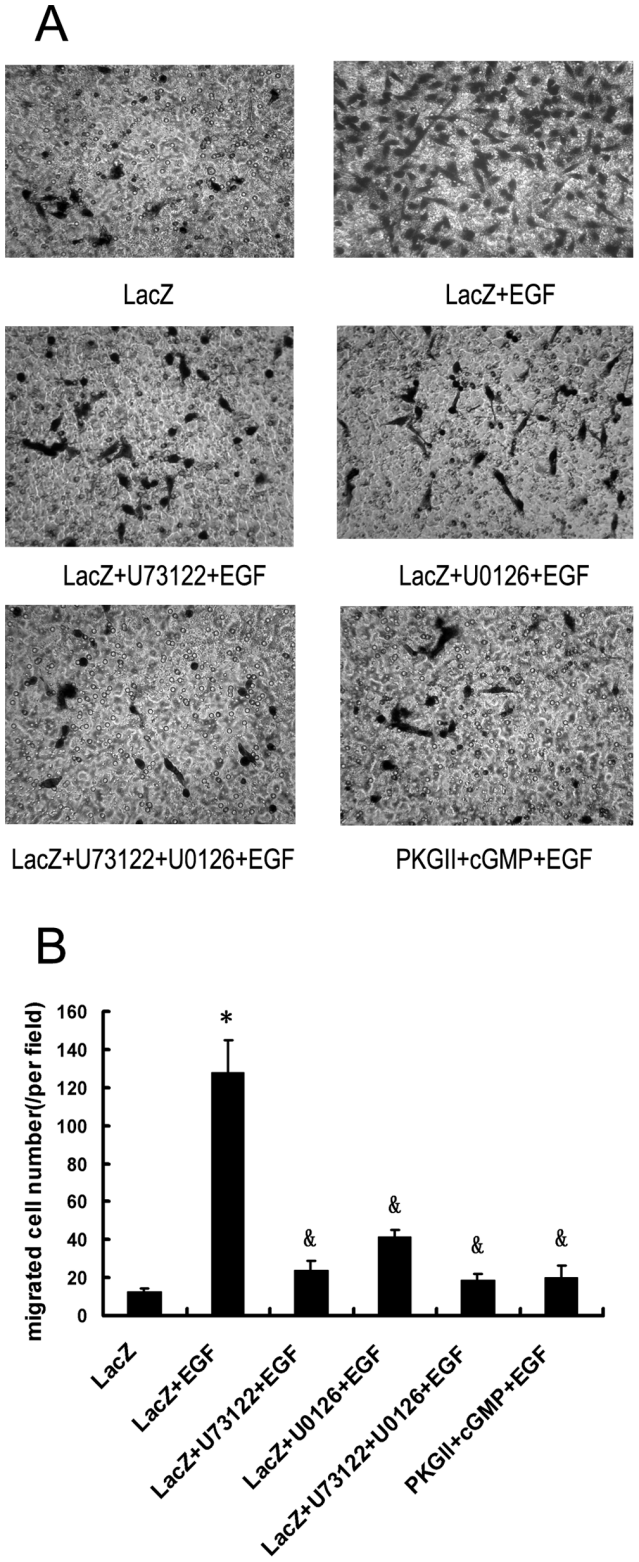
U73122/U1026/PKG II inhibits EGF-induced Cell migration. Migration activity of AGS cells was analyzed with transwell system. The cells were infected with Ad-LacZ or Ad-PKG II for 48 h and serum starved o/n. In Ad-LacZ+EGF and Ad-PKGII+EGF groups, EGF (100 ng/ml) was added to the culture medium; In Ad-LacZ+cGMP+EGF and Ad-PKGII+cGMP+EGF groups, cells were treated with 8-pCPT-cGMP for 1 h and then EGF (100 ng/ml) was added to the culture medium. The migration time was 12 h. A: Representative figures of migrated-cells stained by Giemsa (×200); B: The number of migrated cells in each group. The data shown are the means ± SD from 5 independent experiments, each performed in duplicate (*P<0.01, compared to LacZ group; ^&^P<0.01, compared to LacZ+ EGF group).

Cell migration is important in normal physiology and in disease. Acquisition of migratory ability by cancer cells is a characteristic that contributes to spread of metastatic tumor cells to distant organs. Recent data showed that EGF could stimulate the migration of cancer cells. The mechanism through which EGF stimulates cell migration is not clear but some data indicated that EGF may do this through initiating signal transduction of PLCγ1 and MAPK/ERK-mediated pathways. In this experiment, we detected the migration-stimulating effect of EGF in AGS cells and used inhibitor of key components in the signal pathways to investigate the possible signal transduction associated with the effect. The results showed that EGF treatment increased the migration activity of AGS cells and both MEK (key component of MAPK/ERK-mediated pathway) inhibitor U0126 and PLCγ1 inhibitor U73122 inhibited EGF-induced migration, indicating that EGF stimulated cell migration activity through activating both MAPK/ERK and PLCγ1 mediated signal transduction pathways (Fig. 1).

### PKG II Blocks EGF-induced Tyr 992 and Tyr 1068 Phosphorylation of EGFR

**Figure 2:**
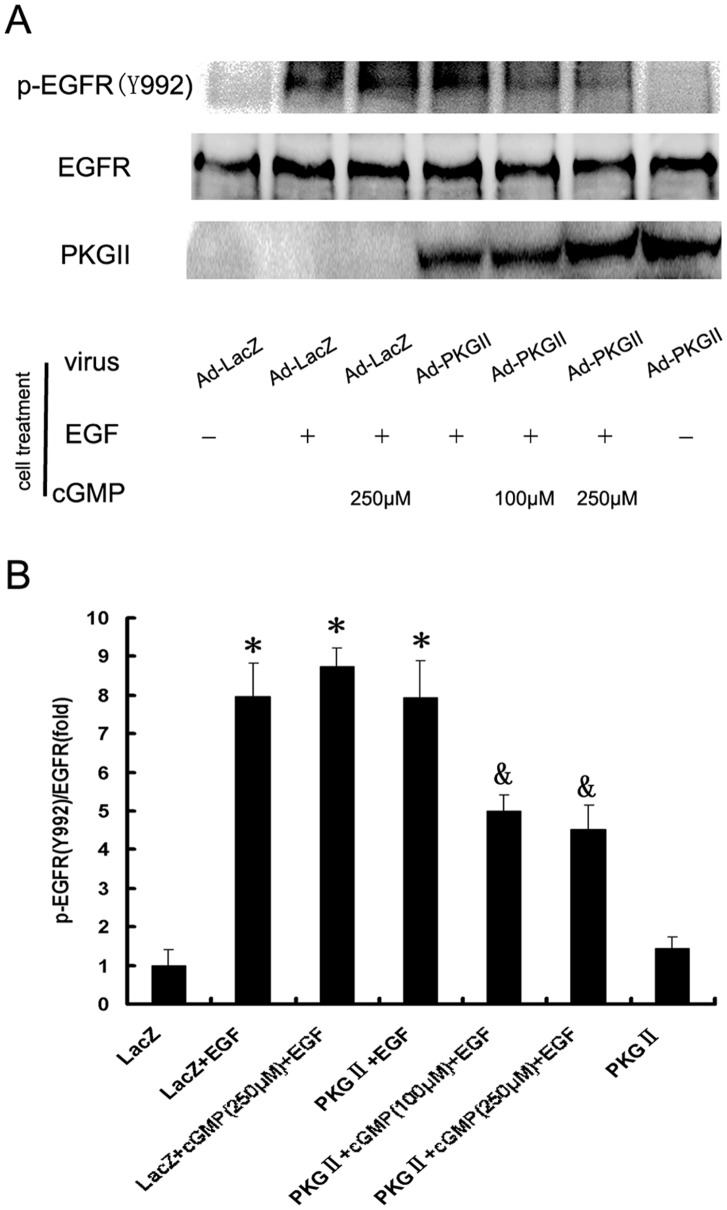
PKG II prevents EGF-induced Tyr 992 phosphorylation of EGFR. AGS cells were infected with Ad-LacZ or Ad-PKG II for 48 h and serum starved o/n. In Ad-LacZ+EGF and Ad-PKGII+EGF groups, cells were incubated with EGF (100 ng/ml) for 5 min. In Ad-LacZ+cGMP+EGF and Ad-PKGII+cGMP+EGF groups, cells were treated with 8-pCPT-cGMP for 1 h and then with EGF (100 ng/ml) for 5 min. Cells were harvested and lysed as described in material and methods. The cell lysate was subjected to Western blotting with antibody against Tyr 992 phospho-EGFR and EGFR. Total EGFR protein levels were used as loading control. Densitometry analysis was performed to quantify the positive bands. A: A representative of initial results of three independent experiments. B: Results of densitometry analysis. The data shown are the means ± SD from 3 independent experiments (*P<0.05, compared to LacZ group and PKG II group; ^&^P<0.05, compared to LacZ+EGF group, LacZ+cGMP(250 µM)+EGF group and PKG II+EGF group).

**Figure 3:**
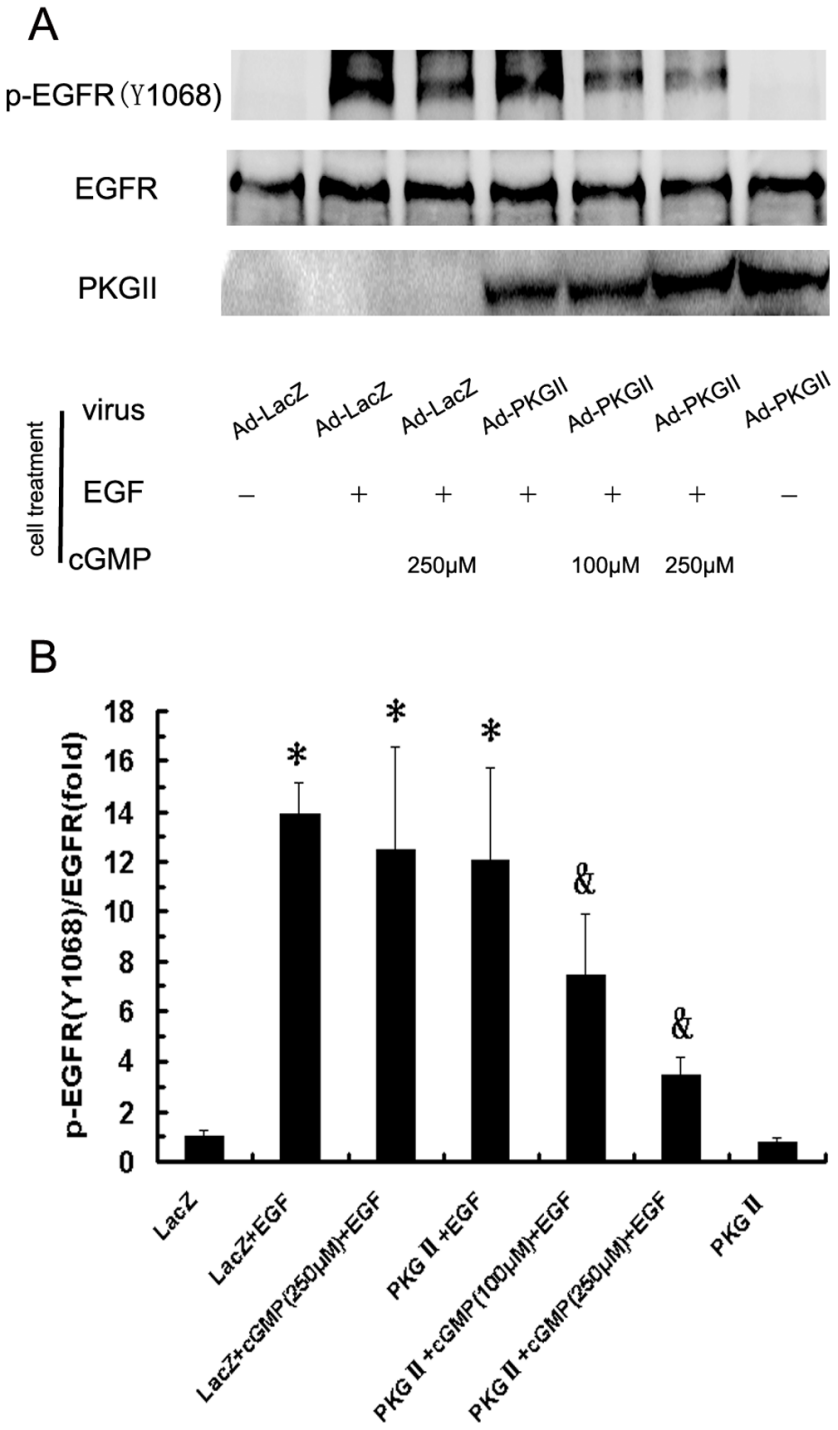
PKG II prevents EGF-induced Tyr 1068 phosphorylation of EGFR. AGS cells were treated same as described in Figure 2. Western blotting was applied to detect Tyr 1068 phosphorylation of EGFR and densitometry analysis was performed to quantify the positive bands. A: A representative of initial results of three independent experiments. B: Results of densitometry analysis. The data shown are the means ± SD from 3 independent experiments (*P<0.05, compared to LacZ group and PKG II group; ^&^P<0.05, compared to LacZ+EGF group, LacZ+cGMP(250 µM)+EGF group and PKG II+EGF group).

When EGF binds with EGFR, it causes auto-phosphorylation of the receptor. There are several auto-phosphorylation sites which are connected to different signal transduction pathway. Tyrosine 992 and Tyrosine 1068 are among the auto-phosphorylation sites of EGFR and are associated with PLCγ1-mediated and MAPK/ERK-mediated signaling respectively. In this experiment, we investigated the inhibitory effect of PKG II on the Tyrosine 992 and Tyrosine 1068 phosphorylation of EGFR in differently treated AGS cells by using Western blotting. The results showed that EGF treatment caused a 14 folds increase of Tyrosine 992 and an 8 folds increase of Tyrosine 1068 phosphorylation of EGFR. In cells infected with Ad-PKG II and stimulated with cGMP, the phosphorylation was significantly decreased (Fig. 2, 3). This indicated that PKG II could prevent EGF-induced Tyrosine 992 and Tyrosine 1068 phosphorylation of EGFR and consequently inhibit PLCγ1-mediated and MAPK/ERK–mediated signaling.

### PKG II Prevents EGF-triggered Main Events of PLCγ1-mediated Signal Transduction Pathway

#### (1) PLCγ1 activation

**Figure 4:**
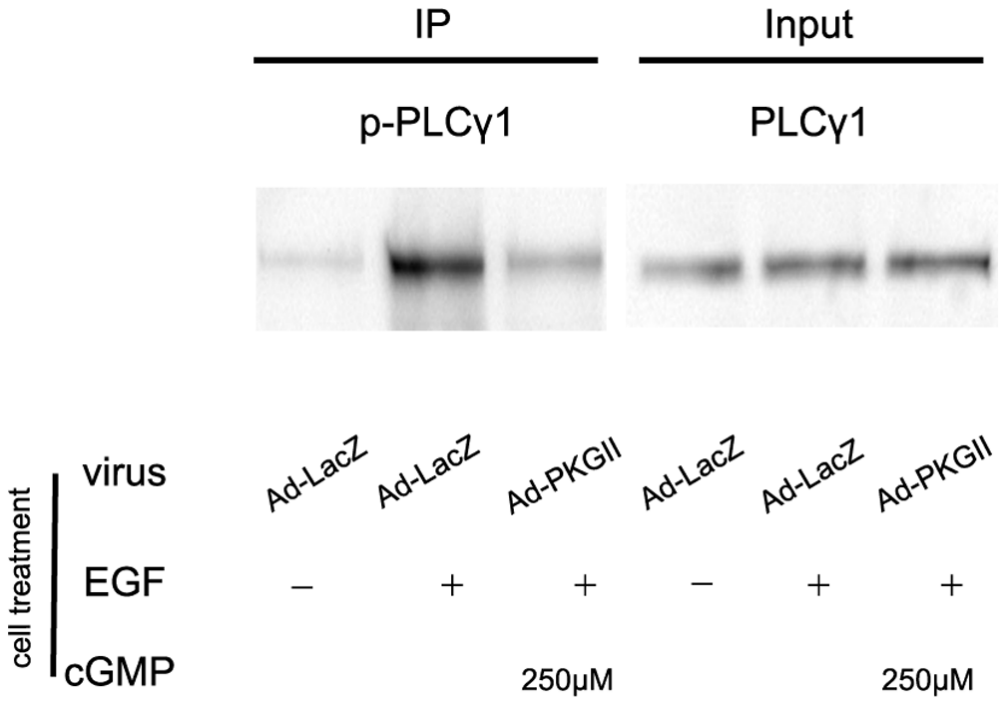
PKG II blocks the phosphorylation of PLCγ1. AGS cells were grown in 100-mm plates and infected with either Ad-LacZ or Ad-PKG II. Then, the cells were serum-starved o/n and treated differently: in Ad-LacZ group, no drug treatment; in Ad-LacZ+EGF group, the cells were incubated with EGF(100 ng/ml) for 5 min; in Ad-PKG II+ cGMP+EGF group, cells were incubated with 250 µM 8-pCPT-cGMP for 1 h and followed by incubating with EGF (100 ng/ml) for 5 min. Immunoprecipitation with antibody against PLCγ1 was performed to precipitate PLCγ1 and the phosphorylation of precipitated PLCγ1 was analyze by Western blotting with antibody against phospho- PLCγ1(Tyr783). The results shown are representative of three independent experiments.

PLCγ is the downstream component of receptor tyrosine kinases (RTKs). It has two isoforms: PLCγ1 is ubiquitously distributed and PLCγ2 is expressed mainly in hematopoietic cells. Activation of PLCγ1 requires its recruitment to the membrane and association, through its SH2 domain, with activated RTKs such as EGFR. This association will result in the phosphorylation of PLCγ1 on tyrosine residues, particularly on tyrosine 783, and an increase of its enzymatic activity. We applied IP method to isolate PLCγ1 and then used Western blot method to detect the phosphorylation of PLCγ1. The results showed that EGF treatment caused an obvious increase of Tyr783 phosphorylation of PLCγ1 and the increase of PKG II activity through infecting the cells with Ad-PKG II and stimulating the cells with cGMP efficiently prevented the EGF-induced phosphorylation of PLCγ1 (Fig. 4).

#### (2) DAG formation

**Figure 5:**
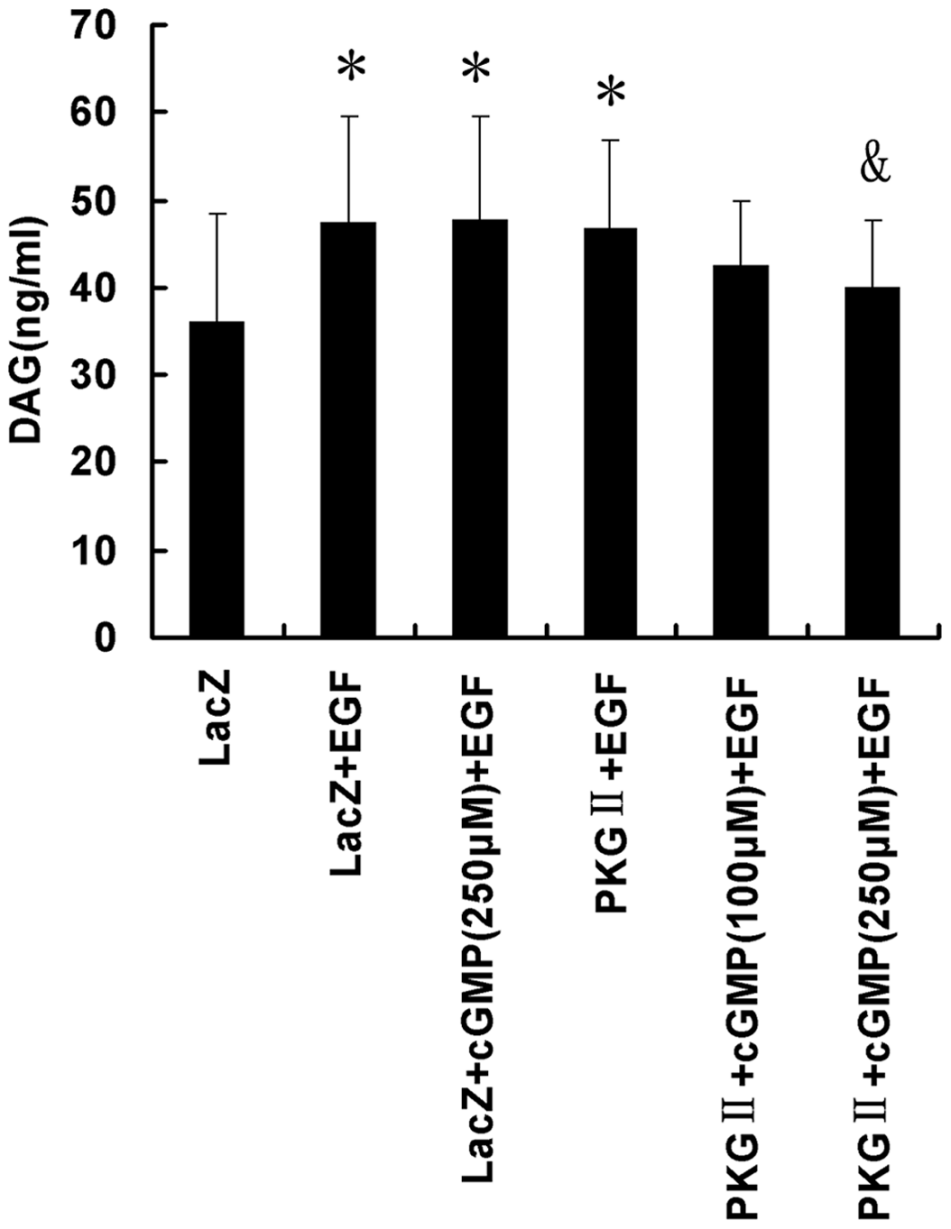
PKG II suppresses the formation of DAG. AGS cells were treated same as described in Figure 4. The concentration of DAG in the cell extracts was measured by ELISA. The data shown are the means ± SD from 5 independent experiments, each performed in duplicate [*P<0.05, compared to LacZ group; ^&^P<0.05, compared to LacZ+EGF group, LacZ+cGMP(250 µM)+EGF group and PKG II+EGF group].

Once it is activated, PLCγ1 can catalyze the hydrolysis of phosphatidylinositol 4,5-bisphos-phate (PI-4,5P_2_) into inositol 1,4,5-trisphosphate (IP_3_) and diacylglycerol (DAG), two molecules that regulate the mobilization of intracellular Ca^2+^ and protein kinase C activity respectively. To observe the effects of EGF and PKG II on the formation of DAG, ELISA method was used to detect DAG concentration in AGS cells. The results showed that in EGF stimulated AGS cells, the level of DAG increased obviously and pre-infection with Ad-PKG II and treatment with 8p-CPT-cGMP inhibited the formation of DAG caused by stimulation with EGF (Fig. 5).

#### (3) Ca^2+^ releasing

**Figure 6:**
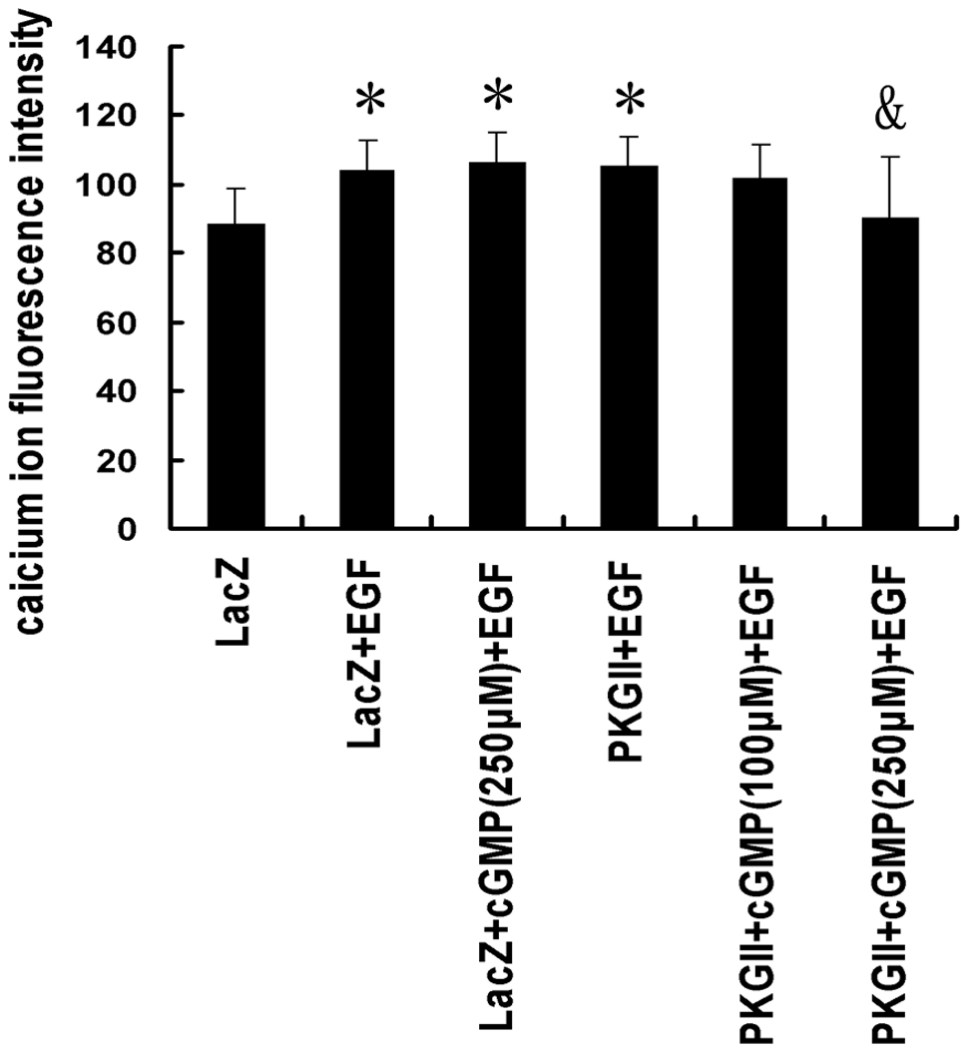
PKG II inhibits the release of Ca^2+^ from the endoplasmic reticulum (ER). Either Ad-PKG II-infected or Ad-LacZ-infected cells growing in a 96-well plate were serum-starved for 12 h, loaded with 5 µM of membrane permeable calcium indicator fluo-3/AM for 30 min at 37°C in DMEM. After loading with the fluo-3/AM, cells were washed with PBS solution and suspended in DMEM, and then incubated with 8-pCPT-cGMP (100 µΜ and 250 µΜ) for 30 min, and then stimulated with EGF (100 ng/ml) for 5 min. Fluorescence measurements were performed using an Olympus Fluoview-500 confocal system. The data shown are the means ± SD from 5 independent experiments, each performed in duplicate [*P<0.05, compared to LacZ group; ^&^P<0.05, compared to LacZ+EGF group, LacZ+cGMP(250 µM)+EGF group and PKG II+EGF group].

IP3 and DAG are second messengers in PLCγ1-mediated signal transduction pathway. IP3 is known to stimulate the release of calcium from internal stores. We used calcium indicator fluo-3/AM to detect calcium in the cytoplasm. The results showed that EGF treatment increased the release of Ca^2+^ from endoplasmic reticulum (ER) to cytoplasm and high expression and activity of PKG II significantly inhibited the release, reversing the effect of EGF treatment (Fig. 6).

#### (4) Activation of PKCα

**Figure 7:**
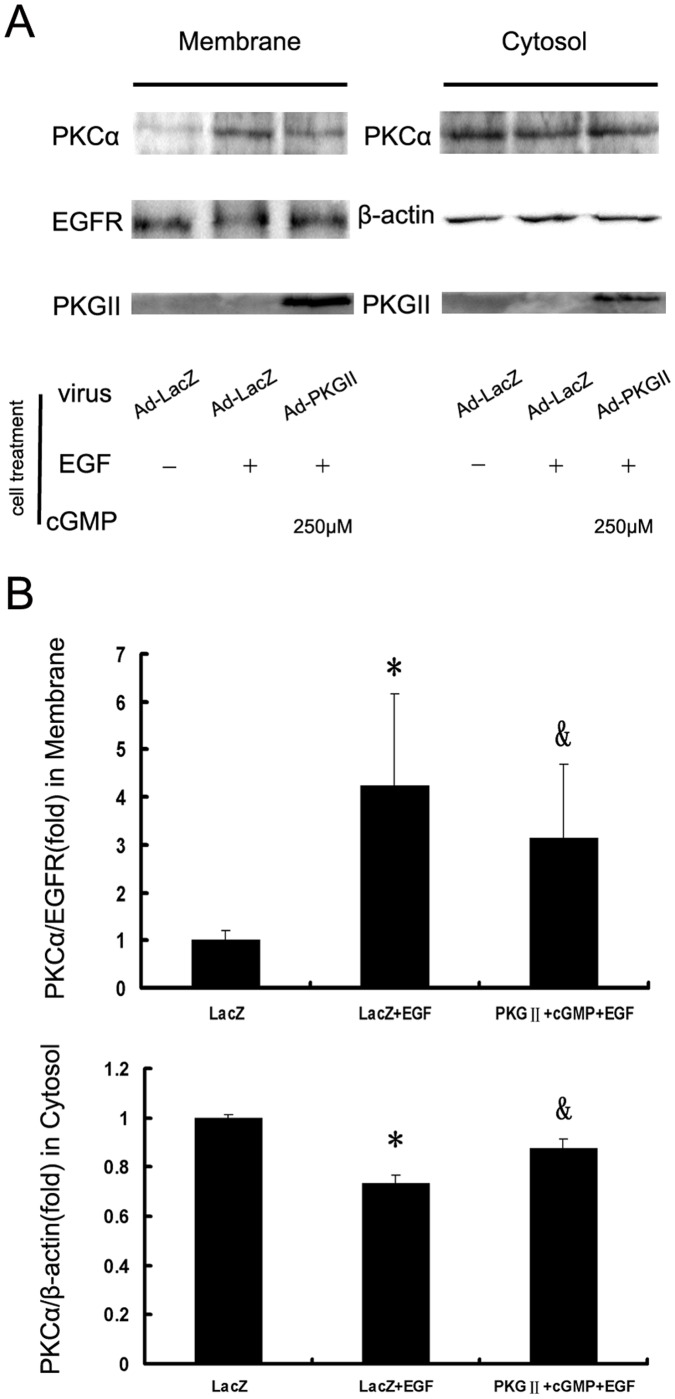
PKG II prevents the activation of PKCα. AGS cells were treated same as described in Figure 4. Subcellular fractionation into cytosol and membrane fractions was performed by using Membrane and Cytosol Protein Extraction Kit. Western blotting was used to detect PKCα either on the membrane or in the cytosol. Densitometry analysis was performed to quantify the positive bands. A: A representative of initial results of three independent experiments. B: Results of densitometry analysis. The data shown are the means ± SD from 3 independent experiments (*P<0.05, compared to LacZ group; ^&^P<0.05, compared to LacZ+EGF group).

PKCα, an isoform of protein kinase C, is a key component of PLCγ1-mediated signal pathway and can be activated by Ca^2+^ and DAG. Being activated, PKCα translocates from cytosol to membrane of the cells. So, the amount of PKCα on the membrane represents the activation of PKCα. In this experiment, we investigated the inhibitory effect of PKG II on the activation of PKCα in differently treated AGS cells by using Western blotting. The results showed that within five minutes after adding the EGF to culture medium, the translocation of PKCα from cytosol to membrane increased dramatically and the translocation was inhibited by pre-infecting the cells with Ad-PKG II and treatment with 8-pCPT-cGMP (Fig. 7).

#### (5) Activation of CaMKIIα

**Figure 8:**
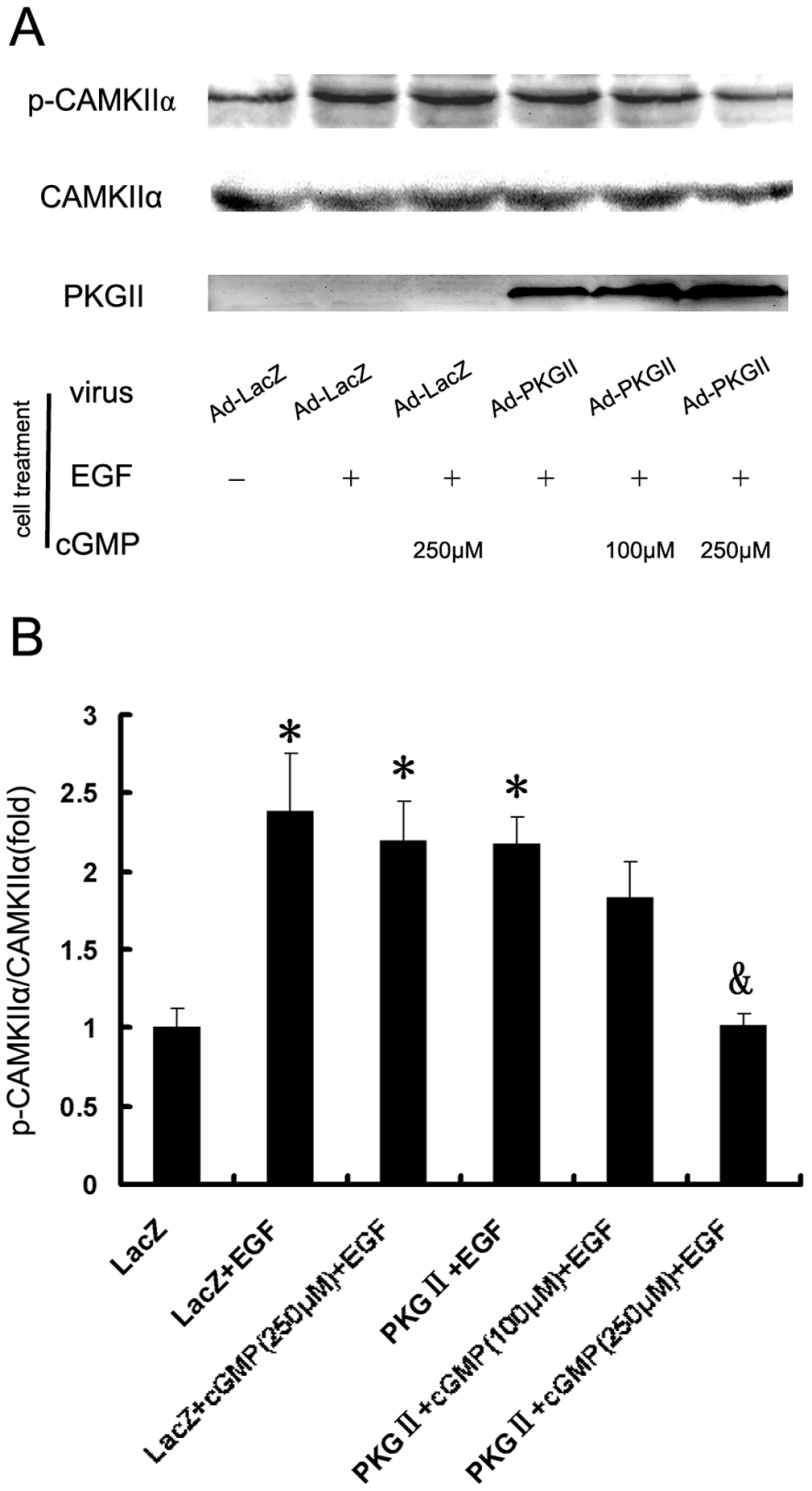
PKG II blocks the phosphorylation of CAMK Iiα. AGS cells were treated same as described in Figure 2. Western blotting was applied to detect the phosphorylation of CAMK IIα. Densitometry analysis was performed to quantify the positive bands. A: A representative of initial results of three independent experiments. B: Results of densitometry analysis. The data shown are the means ± SD from 3 independent experiments (*P<0.05, compared to LacZ group; ^&^P<0.05, compared to LacZ+EGF group, LacZ+cGMP(250 µM)+EGF group and PKG II+EGF group).

Studies on the regulation of Ca^2+^/calmodulin(CaM)-dependent kinase II alpha (CaMKIIα), which is a primary isoform of CaMKII, have suggested that when Ca^2+^/CaM binds with CaMKIIα, intra-molecular autophosphorylation occurs and the phosphorylation maintains the persistent activation of the enzyme. The antibody against phospho-CaMKIIα (Thr286) was applied in Western blotting to detect the phosphorylation of CaMKIIα. The results showed that in AGS cell treated with EGF, Thr286 phosphorylation of CaMKIIα increased by nearly 2.5 folds and infection with Ad-PKG II and treatment with 8-pCPT-cGMP inhibited the increase of the phosphorylation induced by EGF (Fig. 8).

### PKG II Inhibits EGF-induced Activation of the Key Components of MAPK/ERK -mediated Signal Transduction Pathway

#### (1) Activation of MAPK/ERK

**Figure 9:**
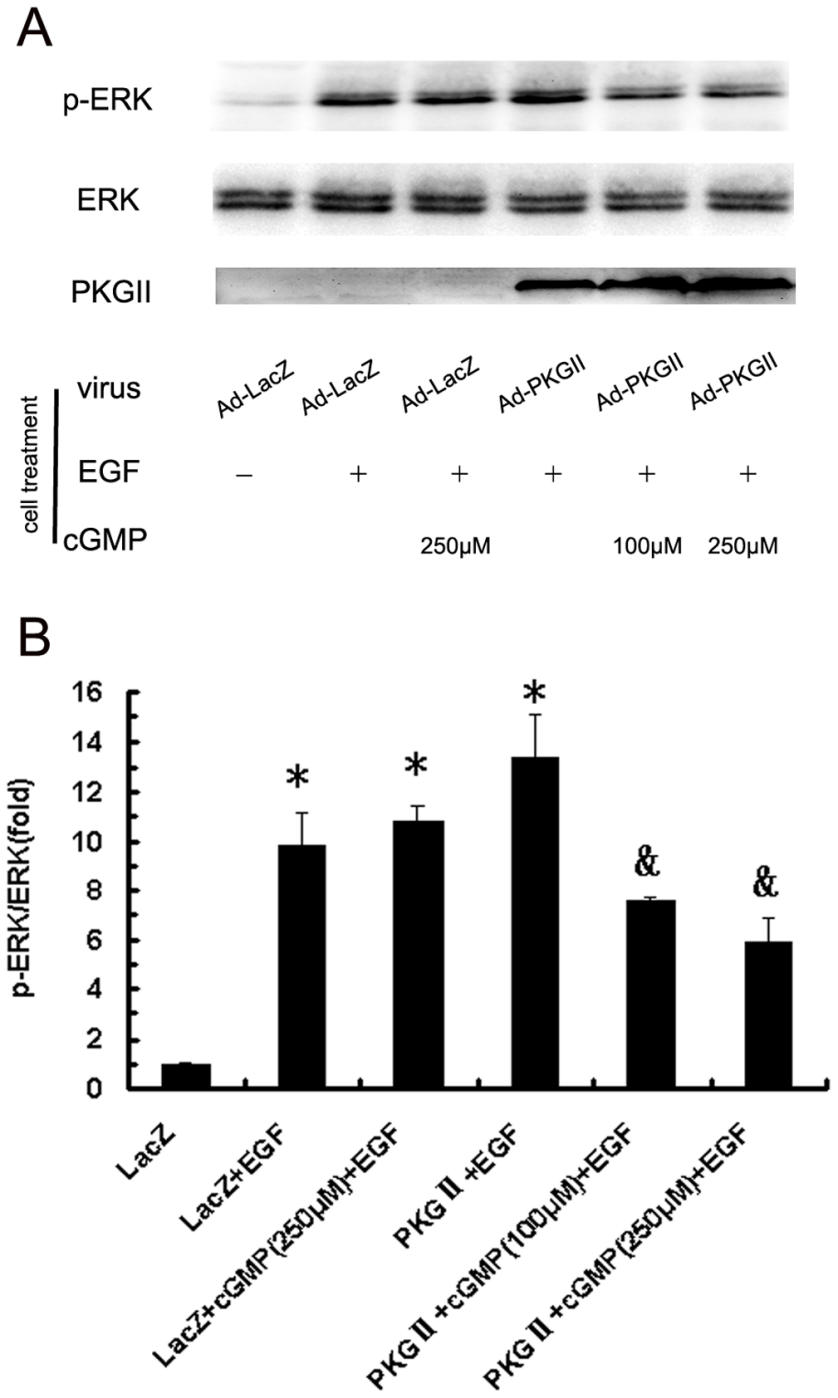
PKG II blocks the phosphorylation of ERK. AGS cells were treated same as described in Figure 2. Western blotting was applied to detect the phosphorylation of ERK. Densitometry analysis was performed to quantify the positive bands. A: A representative of initial results of three independent experiments. B: Results of densitometry analysis. The data shown are the means ± SD from 3 independent experiments (*P<0.05, compared to LacZ group; ^&^P<0.05, compared to LacZ+EGF group, LacZ+cGMP(250 µM)+EGF group and PKG II+EGF group).

MAPK/ERK is the key component of MAPK/ERK-mediated pathway. Phosphorylation at both threonine 202 and tyrosine 204 residues of ERK1 and threonine 185 and tyrosine 187 residues of ERK2 is required for full enzymatic activation. The antibody against p-ERK1/2 (Thr 202/Tyr 204) was applied in Western blotting to detect the dual phosphorylation of ERK. The results showed that within five minutes after adding EGF to cell culture medium, phosphorylation of ERK1/2 in AGS cells increased dramatically (10 folds) and the phosphorylation was inhibited by pre-infecting the cells with Ad-PKG II and activating the enzyme with 8-pCPT-cGMP (Fig. 9).

#### (2) Activation of ras

**Figure 10:**
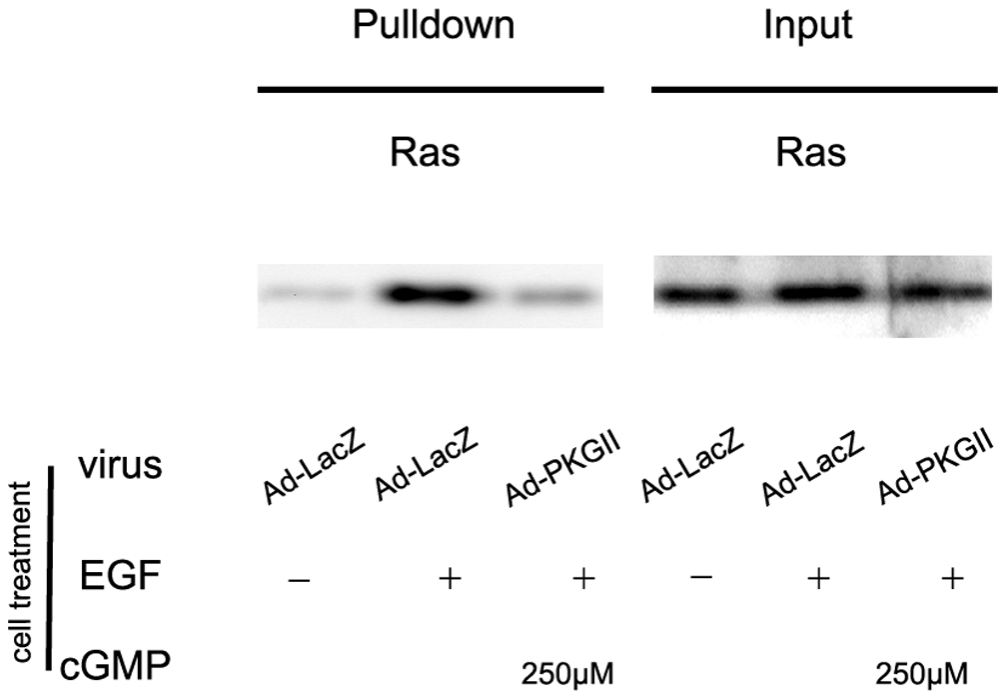
PKG II inhibits EGF-induced Ras activation. The “pull-down” method was used to detect the activated Ras. Cell lysate was prepared and equal amounts of protein were incubated with GST-RBD beads as described in materials and methods. Binding complexes were collected by centrifugation, resolved by SDS-PAGE, transferred onto PVDF membrane and probed with anti-pan Ras antibody. The results shown are representative of three independent experiments.

Small G protein Ras is another key component in MAPK/ERK-mediated signal pathway. It has two forms in cells: GTP-bound active form and GDP-bound inactive form. Once Ras is in GTP-bound form, it can bind and activate Raf-1 and start the consequent activations of serine/threonine kinases in the signal pathway. We applied “pull-down” method to detect the activated Ras. The result showed that after adding EGF to the culture medium, active Ras in AGS cells increased obviously within five minutes. Infecting the cells with Ad-PKG II and stimulating them with 8-pCPT-cGMP before adding EGF significantly prevented the EGF-induced Ras activation (Fig. 10).

### PKG II Inhibits EGF-induced Activation of RAC1

**Figure 11:**
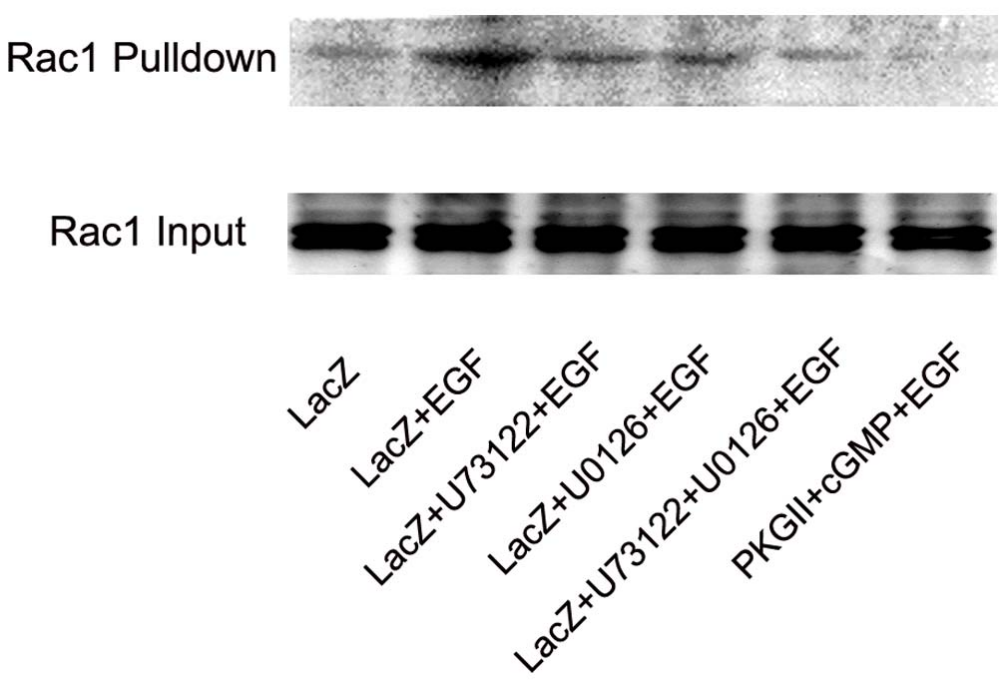
U73122/U1026/PKG II inhibits EGF-induced activation of RAC1. The “pull-down” method was used to detect the activated RAC1. Cell lysate was prepared and equal amounts of protein were incubated with GST-PBD beads as described in materials and methods. Binding complexes were collected by centrifugation, resolved by SDS-PAGE, transferred onto PVDF membrane and probed with anti-pan RAC1 antibody. The results shown are representative of three independent experiments.

Small G protein RAC1 is the main member of Rho family which play important role in regulating migration of cancer cells. Both PLCγ1 and MAPK/ERK mediated signal transduction can activate RAC1 and thereafter stimulate cell migration. To further confirm the inhibitory effect of PKG II on EGF/EGFR-induced signaling which is related to migration, “Pull-down” method was applied to detect the inhibition of PKG II on activation of RAC1. The result showed that EGF treatment caused an obvious increase of active RAC1 and high activity of PKG II efficiently inhibited the activation of RAC1 (Fig. 11). This provided further evidence of the inhibition of PKG II on EGF-induced migration of gastric cancer cells.

### PKGII Interacts with EGFR and Causes Thr-phoshporylation of the Receptor

**Figure 12:**
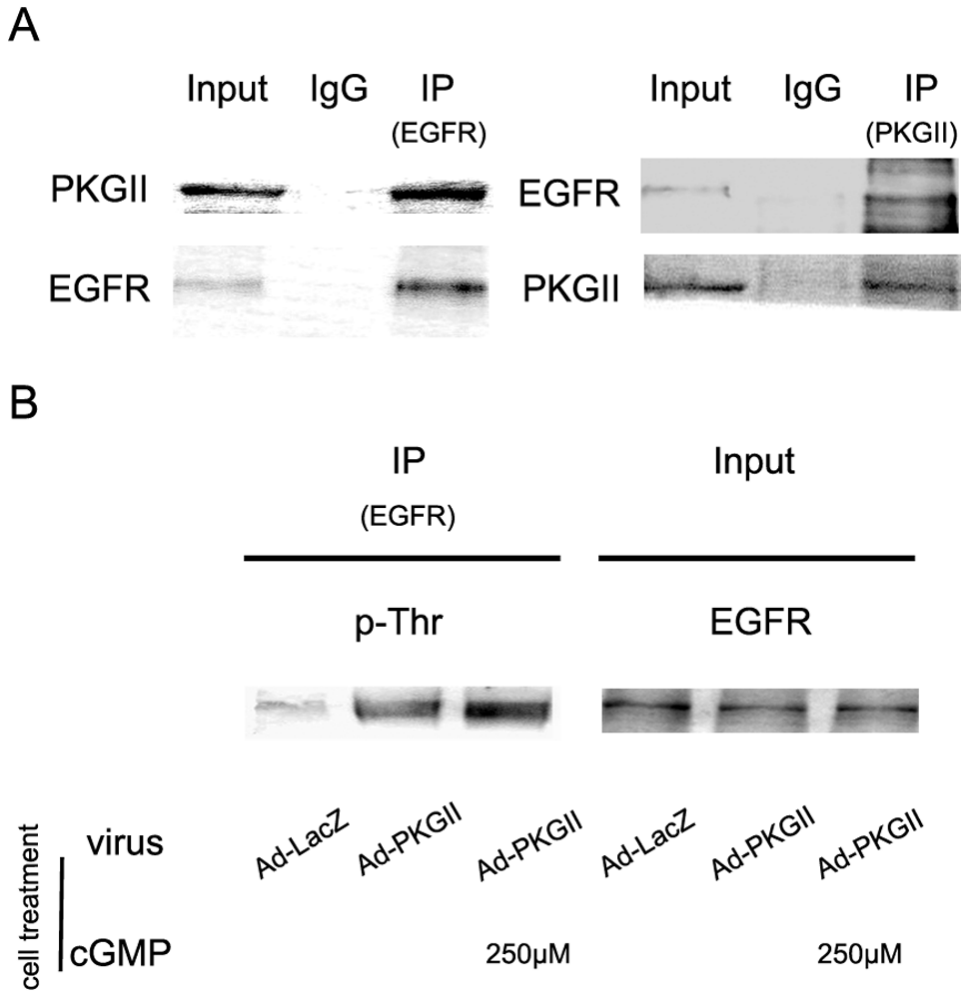
PKG II binds with EGFR and causes Threonine phosphorylation of it. A: Results of Co-immunoprecipitation. AGS cells were grown in 100-mm plates and infected with Ad-PKG II. After being serum-starved o/n, treated with 250 µM 8-pCPT-cGMP for 1 h, and incubated with EGF (100 ng/ml) for 5 min, the cells were lysed and the lysate was immunoprecipitated with anti-PKG II antibody or isotype-matched IgG. The precipitates were probed with anti-EGFR antibody. Five percentage of cell lysate was used as a protein input control. The contrary experiment, i.e. immunoprecipitated with anti-EGFR antibody and probed with anti-PKG II antibody, was also performed. B: Results of immunoprecipitation and Western blotting. AGS cells were treated same as A, and the lysate was immune-precipitated with antibody against EGFR to enrich the protein. The precipitates were subjected to Western blotting with pan anti-Threonine phosphorylation antibody. The results shown are representative of three independent experiments.

The mechanism through which PKGII blocks the EGF-induced activation of EGFR was preliminarily investigated in this experiment. Co-Immunoprecipitation was applied to detect the interaction between PKGII and EGFR. Western blotting with pan anti phosphorylation of threonine antibody was used to detect the Threonine phosphorylation of EGFR caused by PKGII. The results of Co-Immunoprecipitation showed that in AGS cells infected with Ad-PKG II and stimulated with 8-CPT-cGMP, direct binding between PKG II and EGFR was detected (Fig. 12, panel A). Results of Western blotting showed that activation of PKG II caused threonine phosphorylation of EGFR (Fig. 12, panel B). This indicated that PKG II blocked the activation EGFR through binding with the receptor and causing phosphorylation of it.

## Discussion

Currently, two cGMP-dependent protein kinases (PKGs), PKG I and PKG II, have been identified in mammalian cells [10], [11]. PKG I is widely distributed within the body and owing to its inhibiting effect on tumor growth and invasiveness and inducing effect on apoptosis of tumor cells, it has been identified as a tumor suppressor [12]. The expression of PKG II is more tissue-restricted [13]. For a long time, in contrast to the well-proved anti-tumor effect of PKG I, no research data clearly indicated antitumor role of PKG II and this kinase was only implicated in several physiological functions including intestinal secretion, bone growth, and learning and memory [14]. However, research interest about PKG II is increasing and some new functions of PKG II have been found recently, including the role of PKG II in regulation of epithelial sodium channel and mechano-signal transduction [15]–[17]. More importantly, accumulating research data indicated that PKG II was related to proliferation and apoptosis in some cells, especially in tumor cells, strongly suggesting the potential role of this enzyme in regulating biological activities of tumor cells [1]–[6].

EGFR exists on the surface of all cells. With a molecular weight of 170KD, EGFR has an extracellular domain, a cross-membrane domain and an intracellular domain. The intracellular domain of EGFR has 542 amino-acid residues and can be divided into approximate membrane sub-domain, tyrosine kinase sub-domain, and C-terminal sub-domain [18]. The activating process of EGFR includes the tyrosine phosphorylation of its intracellular domain and different phosphorylation sites on the domain are related to different signal pathways. When EGFR is activated, it can recruit effector proteins to its phosphorylated C-terminal sub-domain and initiates the effector protein-mediated pathways [19], [20]. Among the phosphorylation sites, tyrosine 1068 and 1086 are related to MAPK/ERK-mediated pathway and tyrosine 992 and 1173 are related to PLCγ-mediated signal pathway [7], [8]. Our previous results showed that PKG II could inhibit EGF-induced tyrosine 1068 phosphorylation of EGFR in gastric cancer cell line BGC-823 [6], raising the question whether PKG II can inhibit the phosphorylation of other tyrosine sites on EGF/EGFR and thereafter have a wide-range inhibition on EGF/EGFR-induced signal transductions and related biological activities of gastric cancer cells.

In this paper, we investigated the action of PKG II on EGF-induced migration activity of gastric cancer cell line AGS. The result showed that PKG II had significant inhibition on cell migration caused by EGF. This provides further evidence for revealing the tumor inhibitory effect of PKG II. Research data have shown that among the EGF/EGFR initiated signal transduction pathways, PLCγ1 and MAPK/ERK mediated signal transduction pathways are related to migration activity [21], [22]. To confirm this in gastric cancer cells, we used inhibitor of signal transduction component to identify the participation of MAPK/ERK and PLCγ1-mediated pathways in the process. The results showed that MEK inhibitor U0126 and PLCγ1 inhibitor U73122 partially blocked EGF/EGFR-induced migration activity of AGS cells, indicating that both signal transduction pathways participated in the regulating process. To elucidate the potential inhibitory action of PKG II on these signal transduction pathways, we first explored the inhibitory effect of PKG II on EGF initiated PLCγ1-mediated signal transduction pathway. The results showed that PKG II prevented all of the main events in this signal transduction pathway, including the Tyr 992 phosphorylation/activation of EGFR, the phosphorylation/activation of PLCγ1, the formation of second messenger DAG, the release of calcium into cytoplasma, and the activation of PKC and CAMK IIα. For the inhibition of PKG II on EGF/EGFR induced MAPK/ERK-mediated signal transduction pathway, our previous work have shown that PKG II inhibits the activation of all key components in the pathway induced by EGF in gastric cancer cell line BGC-823 [6]. In this paper, we investigated the inhibitory effect of PKG II on EGF/EGFR-induced activation of key components in this pathway. The results confirmed that PKG II inhibited EGF-induced activation of Ras protein and MAPK/ERK in AGS cells, suggesting that PKG II also inhibited EGF/EGFR-induced signal transduction of MAPK/ERK-mediated pathway in this cancer cell line. These results systematically revealed that PKG II inhibited EGF-induced migration of gastric cancer cells through blocking EGF/EGFR initiated signal transduction of PLCγ1 and MAP/ERK-mediated pathways.

The signal transduction of both PLCγ1 and MAP/ERK-mediated pathways can activate small G protein Rac1, which is a key component in regulating cell migration [23], [24]. To confirm the inhibition of PKG II on this important event in EGF-induced migration of GAS cells, we applied “pull-down” method to check the activity of Rac1 in differently treated AGS cells. The results showed that during EGF-induced migration, Rac1 was activated and activation was related to both PLCγ1 and MAP/ERK-mediated signaling. Furthermore, PKG II inhibited the EGF-induced activation of Rac 1. This further confirmed the inhibitory effect of PKG II on EGF/EGFR initiated cell migration.

EGFR is closely associated with tumorigenensis. Over expression and mutation of EGFR often happen in most cancers. Research data showed that over 50%–70% of lung cancer, colon cancer and breast cancer have high expression of EGFR [25]. Furthermore, cancer patients with over-expression of EGFR usually have poor prognosis. For example, EGFR over-expression was detected in 60% of non-small cell lung cancer (NSCLC) patients and the prognosis of the patients were poor, with a survival of 4–5 months only [26]. *In vitro* experiments confirmed that over-expression of EGFR caused transformation of NIH-3T3, Rat-1 and NRK cells and blocking EGFR activation inhibited proliferation of some tumor cells [27]. Consequently, EGFR is the first growth factor receptor taken as cancer therapy target. Several methods of inhibiting EGFR activity and related signal transduction, including specific antibodies against EGFR and inhibitors of EGFR, received intensive research [28]–[30]. New and more effective methods in blocking EGFR-mediated signal transduction will be helpful in cancer therapy. Therefore, the finding that PKG II can inhibit the activation of EGFR and the consequent signal transduction has important significance. It strongly suggests that PKG II is a potential endogenous EGFR inhibitor and will provide new hint on strategy of cancer therapy.

Research data showed that some protein kinases can cause phosphorylation of EGFR and affect its activation and/or destination. For example, protein kinase C (PKC) can cause the phosphorylation of Threonine 654 of EGFR and regulates receptor binding and internalization [31]. Serine 1046/1047 (Ser1046/1047) phosphorylation site is required for EGFR desensitization in EGF-treated cells [32].In our experiments, we detected the phosphorylation of Thr654 and Ser1046/1047 of EGFR and found that PKG II did not cause the phosphorylation of these phosphorylation sites. However, our results showed that there was a direct interaction between PKG II and EGFR and PKG II caused Threonine phosphorylation of EGFR. This indicated that PKG II blocked the activation of EGFR through binding with and phosphorylating the receptor. The exact phosphorylation site will be our next research goal.

## Materials and Methods

### Cell Line and Reagents

Human gastric cancer cell line AGS was provided by the Institute of Cell Biology (Shanghai, China). Adenoviral vectors encoding β-galactosidase (pAd-LacZ) and PKG II (pAd-PKG II) were kind gifts from Dr. Gerry Boss and Dr. Renate Pilz in University of California, San Diego, U.S.A. Dulbecco’s Modified Eagle’s Media (DMEM) and fetal bovine serum (FBS) were from GIBCO (Grand Island, NY). The antibody against PKG II was from ABGENT Biotechnology (San Diego, CA). Goat anti-β-actin, mouse anti-pan-Ras, and mouse anti-PLCγ1 antibodies were from Santa Cruz (Santa Cruz, CA). Rabbit anti-p-EGFR (Tyr1068), rabbit anti-p-EGFR (Tyr992), rabbit anti-EGFR, mouse anti-p-ERK(Thr 202/Tyr 204), rabbit anti-ERK and rabbit anti-p-PLCγ1(Tyr783) antibodies were from Cell Signaling Technology (Danvers, MA). Rabbit anti-PKCα and rabbit anti-p-CaMK IIα (Thr286) were from Bioworld Technology (St. Louis Park, MN). Rabbit anti-p-Thr antibody was from Abcam (MA, USA).Horseradish peroxidase (HRP)-conjugated secondary antibodies were from Jackson Immuno Research Laboratories (West Grove, PA). The cellular permeable cGMP analog 8-pCPT-cGMP was from Calbiochem (San Diego, CA). EGF, U73122 and U0126 were from Sigma (St. Louis, MO). Electrochemiluminescence (ECL) reagents were from Millipore (Billerica, MA). Ca^2+^ indicator Fluo-3/AM and Membrane and Cytosol Protein Extraction Kit were from Beyotime (Jiangsu, China). Human diacyl glycerol (DAG/DG) ELISA Kit was from Cusabio Biotech Co., Ltd. (Newark, DE). All other reagents used were of analytical grade.

### Preparation of Adenoviral Vectors

293A cells were transfected with adenoviral vector encoding LacZ and PKG II respectively and cultured for up to ten days until CPE was seen. The cells and the culture medium were harvested and underwent three freezing-thawing cycles. The supernatant containing adenoviruses (Ad-LacZ and Ad-PKG II ) were used to infect new 293A cells to amplify adenoviruses. The amplified adenoviral preparations were titrated and the pfu/ml was determined, and kept in −80°C until use.

### Cell Culture and Infection with Adenoviral Vectors

AGS cells were cultured in DMEM supplied with 10% FBS and maintained at 37°C in a humidified incubator with 95% air and 5% CO_2_. The medium was changed every two days and the cells were sub-cultured at confluence. On the day before infection, cells were freshly planted at 70–80% confluence, and the infection with Ad-LacZ and Ad-PKG II was performed.

### Western Blotting

Protein samples were subjected to SDS-PAGE (8–12%) gel according to the molecular size of target protein, and electrophoresis and membrane transfer was performed following the manufacturer’s protocol (Bio-Rad, Hercules, CA). The primary antibodies were incubated over night at 4°C in TBS-T (2% Tween-20), and the corresponding secondary antibodies were incubated for 1 h at RT in TBS-T (2% Tween-20), with three washes after each incubation. ECL reagents were used to show the positive bands on the membrane. To perform densitometry analysis, digital images of the positive bands were obtained with Chemidoc XRS and analyzed using the image analysis program Quantity One (Bio-Rad, Hercules, CA, USA). The results were showed as the ratio of target protein/loading control.

### “Pull-down” Analysis of Active Small G protein Ras and Rac1

The activity of Ras was detected with Pull-down method as described previously [9]. In brief, cells growing on 100-mm culture plate were washed three times with cold PBS and lysed by adding 400 µl of the lysis buffer (25 mM HEPES pH 7.5, 150 mM NaCl, 1% NP40, 10% glycerol, 25 mM NaF, 10 mM MgCl_2_, 0.25% sodium deoxycholate, 1 mM EDTA, 1 mM Na_3_VO_4_, 10 µg/ml aprotinin, and 10 µg/ml leupeptin). The sample was collected and centrifuged (14000 g, 4°C, 10 min) to get rid of the debris. The supernatant was incubated with glutathione-Sepharose beads and glutathione *S*-transferase-Ras-RBD (GST-RBD) at 4°C for 1 h. The beads were washed 3 times with lysis buffer and heated in boiled water to release proteins. The protein samples (containing active Ras) were analyzed by Western Blotting with antibody against pan-Ras. The active Rac1 was detected with similar method but with GST-Pak1 protein binding domain (GST-PBD) and antibody against Rac1.

### Immunoprecipitation

The cells growing on 100-mm culture plate were washed two times with cold PBS and lysed by adding 1 ml RIPA buffer (50 mM Tris-HCl pH 7.4, 1% Triton X-100, 1 mM EDTA, 1 mM leupeptin, 1 mM phenylmethylsulfonyl fluoride, 10 mM NaF, 1 mM Na_3_VO_4_) per plate. Antibodies against PLCγ1 and p-PLCγ1 were used for immunoprecipitation. The precipitates were probed with antibodies against target proteins.

### Analysis of Calcium in Cytoplasma

To monitor the effect of EGF and PKG II on EGF-induced calcium release, AGS cells were loaded with 5 µM of membrane permeable calcium indicator fluo-3-acetoxymethyl (AM) ester for 30 min at 37°C in DMEM. After the loading, cells were washed with PBS and suspended in DMEM. Fluorescence measurements were performed using an Olympus Fluoview-500 confocal system. Fluo-3 was excited by argon laser light at 488 nm and fluorescence was measured at wavelengths of 515 nm.

### ELISA Assay of DAG

DAG concentrations were measured by ELISA, according to the manufacturer’s instruction. This assay employs the quantitative sandwich enzyme immunoassay technique. Antibody specific for DAG has been pre-coated onto a micro-plate. Standards and samples are pipetted into the wells and any DAG present is bound by the immobilized antibody. After removing any unbound substances, a biotin-conjugated antibody specific for DAG is added to the wells. After washing, avidin conjugated Horseradish Peroxidase (HRP) is added to the wells. Following a wash to remove any unbound avidin-enzyme reagent, a substrate solution is added to the wells and color develops in proportion to the amount of DAG bound in the initial step. The color development is stopped and the intensity of the color is measured.

### Subcellular Fractionation

Subcellular fractionation into cytosol and membrane fractions was performed by using Membrane and Cytosol Protein Extraction Kit, according to the manufacturer’s instruction. Monolayer cultures were washed three times with ice-cold PBS solution and scraped into cold homogenization buffer (HB) containing 20 mM Tris–HCl (pH 7.4), 4 mM EDTA, 2 mM EGTA, 10% glycerol, 10 g/ml leupeptin, and 1 mM PMSF. The cells were lysed via sonication with 4–15 s intervals and complete lysis was monitored microscopically. The homogenate was ultracentrifuged at 86,000 g for 45 min at 4°C, and the supernatant was designated as the cytosolic fraction. The pellet was resuspended in HB containing 1% Triton X-100 and incubated on ice for 30 min. The samples were then centrifuged at 14,000 g for 20 min at 4°C, and the supernatant was designated as the membrane fraction. All samples were boiled and cleared by centrifugation.

### Cell Migration Assay

Migration activity of AGS cells were detected by transwell system (BD BioCoatTM Control 8.0 mm PET Membrane 24-well Cell culture inserts, BD Biosciences). After trypsinization, 5*10^4^cells were seeded into the upper chamber containing culture medium without FBS. Cell migration to the bottom side of membrane was induced by medium containing 10% FBS in the lower chamber for 12 h at 37°C in a tissue culture incubator. Migrated cells on the bottom side of the membrane were fixed in 40% paraformaldehyde solution for 30 min, stained in Giemsa solution for 10 min, and then rinsed in water. The stained cells were subjected to microscopic examination under a light microscope. Migrated cells were counted in 5 randomly selected fields per insert, and the values were averaged. All experiments were performed with three replicates under each migration condition.

### Statistical Analysis

The data were expressed as means ± standard deviation (SD). Statistical analysis was performed using a two-tailed ANOVA with SPSS statistical software. A *P*-value of less than 0.05 was considered significant.
